# Gynecomastia in HIV-positive adult men receiving efavirenz-based antiretroviral therapy at Newlands clinic, Harare, Zimbabwe

**DOI:** 10.1186/s12879-019-4332-5

**Published:** 2019-08-13

**Authors:** Sandra Shawarira-Bote, Tinei Shamu, Cleophas Chimbetete

**Affiliations:** Newlands Clinic, Harare, Zimbabwe

**Keywords:** Efavirenz, Gynecomastia, HIV, Incidence

## Abstract

**Background:**

Gynecomastia is known to occur in some men taking an efavirenz-based antiretroviral therapy (ART) regimen. However, the incidence and outcomes of gynecomastia are not known in Zimbabwe. We described the characteristics and outcomes of gynecomastia among male patients on an efavirenz-based ART regimen.

**Methods:**

We conducted a retrospective cohort review of data of all male patients aged ≥18 years taking an efavirenz-based regimen at Newlands Clinic, Harare, Zimbabwe before 31 March 2017. The primary outcome was gynecomastia as defined by breast/nipple enlargement reported by patient and confirmed by clinical palpation. Routinely collected data on demographics, baseline CD4, body mass index, duration on efavirenz, clinical presentation and outcomes were extracted from the clinic database and analysed using STATA 12.1. We investigated for any associations with concomitant medicines using cox regression.

**Results:**

We analysed data for 1432 men with a median age of 40 years (IQR: 33–48). Half of the patients were in WHO stage 1 at ART commencement. Median body mass index and CD4 count at efavirenz commencement was 21 (IQR: 19–23) and 260 cells/mm^3^ (IQR: 126–412) respectively. The incidence of gynecomastia was 22/1000 person-years (IQR: 17.3–27.8). Over half of the cases (58%) were bilateral and 75% of all cases developed within two years of starting efavirenz. There were no significant associations with concomitant use of isoniazid (HR: 0.95, *p* = 0.87) or amlodipine (HR: 0.43, *p* = 0.24). Gynecomastia resolved in 83.5% of cases following withdrawal of efavirenz with a median time to resolution of 3 months (IQR: 2–9).

**Conclusion:**

The incidence of gynecomastia among patients taking efavirenz-based ART was low with most cases developing early on during treatment. Most cases resolved completely after withdrawing efavirenz.

## Background

The widespread availability of antiretroviral therapy (ART) has seen more people receiving treatment who are infected with the Human Immunodeficiency Virus (HIV). In resource-limited settings, the majority receive a fixed daily dose of efavirenz-based ART as recommended by the World Health Organization (WHO) [1]. Current HIV guidelines recommend ART to all people with HIV, to reduce mortality and morbidity [1]. However, toxicity in stable patients on long-term ART is of concern, with the potential to mitigate against the gains made. Gynecomastia has been recognized as an adverse event to efavirenz. It is defined as breast enlargement due to benign proliferation of glandular tissue [2] and an association with efavirenz-based ART has been demonstrated by several studies [3–5]. The exact mechanism underlying gynecomastia remains unclear, but there are several hypotheses most of which are to do with estrogen hormone activation [5–7]. Besides efavirenz, other drugs have also been associated with gynecomastia [8]. Although benign, gynecomastia can cause significant psychological morbidity with potential negative implications on adherence to ART [9].

Very few studies on efavirenz-related gynecomastia have been done in sub-Saharan Africa, and most of the literature is in form of case reports and case series [4, 6, 9–11]. The incidence of gynecomastia in a study from a high income setting has been reported to be 8/1000 person-years [12], whilst the incidence in African settings is unknown. In South Africa, gynecomastia was a common adverse event reported to National HIV and Tuberculosis Health Care Hotline between June 2013 and July 2014 [4]. In a cross-sectional study done in Malawi, gynecomastia was found to be common (6%) and associated with adverse psychological effects [9] and this was similar to two other small case series (2–6 patients each) which were done in Malawi and Nigeria [3, 13].

There are no local published data on the burden of gynecomastia among patients taking efavirenz-based ART in Zimbabwe. The aim of the study was to determine the incidence of gynecomastia and to describe the clinical characteristics and outcomes in HIV-positive adult men taking efavirenz as part of their ART at Newlands Clinic in Harare, Zimbabwe.

## Methods

### Study design

We conducted a retrospective cohort analysis using routinely collected data of patients recruited into care and commenced on efavirenz-based ART at Newlands Clinic between 1 February 2004 and 31 March 2017. Baseline was defined as the date of efavirenz-based ART commencement. Participants were followed up until diagnosis of gynecomastia, death, loss to follow-up, stopping efavirenz or the 31 March 2017.

### Study setting and participants

Newlands Clinic is an urban HIV treatment center in the capital city of Harare, that offers comprehensive ART at no cost to the patient. The details of Newlands Clinic operations have been described elsewhere [14]. The Clinic had 4769 adult patients aged ≥18 years in care as of 31 March 2017, with the majority (64%) being on a first-line efavirenz-based ART regimen. Adult males constituted 46.8% (*n* = 2231) of the total patient population at Newlands Clinic. During the study period, the preferred first line ART regimen for adult patients changed from stavudine/lamivudine/nevirapine during 2004–2007 to zidovudine/lamivudine/nevirapine until 2010. In 2010 patients were switched to tenofovir/lamivudine/nevirapine until 2013, when they were put on tenofovir/lamivudine/efavirenz until the time of data analysis. Efavirenz was part of the alternative regimen throughout the study period before it became preferred (2013). Patients who would have failed first line ART received two nucleoside reverse transcriptase inhibitors plus a ritonavir-boosted protease inhibitor which was initially lopinavir until 2013 when atazanavir became the preferred drug. Patient records were kept in the Clinic’s electronic database used for routine patient care.

Records of all men (≥18 years) who started efavirenz on or before 31 March 2017 were abstracted from the medical records on the 25th of September 2017. Participants with conditions that were known to cause gynecomastia (chronic liver disease, hyperthyroidism, hyperprolactinemia and hypogonadism) were excluded from this analysis. Concomitant use of medicines previously reported to cause gynecomastia were recorded and associations were assessed. Only the commonly used medicines were sought, and these were spironolactone, isoniazid, methyldopa and amlodipine.

The diagnosis of gynecomastia was made after a patient complained of breast enlargement and this was confirmed clinically by palpation (by the doctor) of a ridge of glandular tissue symmetrically distributed around the areolar complex with varying degrees of tenderness. It could either be unilateral or bilateral. Management of gynecomastia included prompt withdrawal of efavirenz and replacing it with either nevirapine or a protease inhibitor. Resolution was documented when the glandular proliferation was no-longer palpable, and this was indicated in the medical records by an end-date to the diagnosis of gynecomastia.

### Data analysis

Records of all eligible HIV-infected adult men taking efavirenz-based ART between the 1st of February 2004 and the 31 March 2017 were extracted. The following demographic and clinical baseline characteristics were analysed: age, body mass index (BMI), clinical WHO stage and CD4 count at commencement of efavirenz-based ART. BMI data did not meet the quality control standards and therefore we did not include it in our analysis.

Baseline was defined as the day of commencing an efavirenz-based ART regimen. The following characteristics were described in those who developed gynecomastia: duration of exposure to efavirenz (months), clinical features of gynecomastia, management and outcomes.

The study variables were abstracted using SQL Server Management Studio 2010, exported to Microsoft Excel, and then imported into Stata 12.1 (StataCorp) for analysis.

We described numerical characteristics using medians (interquartile ranges, IQR). Proportions were used to describe categorical data (outcomes and clinical presentation). We used the Poisson regression model to calculate the incidence rate and expressed this per 1000 person-years with 95% confidence interval (CI). We used univariate analysis using Cox regression to asses for associations of gynecomastia with concomitant drugs previously reported to be associated with gynecomastia. Medicines were considered to be significantly associated with gynecomastia when the *p*-value was < 0.05. The following medicines were included in the analysis: methyldopa, amlodipine, spironolactone and isoniazid.

### Ethical approval

The study was approved by the Newlands Clinic Scientific Committee and Medical Research Council of Zimbabwe (MRCZ/E/166). The data was de-identified before extraction. No further consent from participants was required since secondary data was used. Gynecomastia was managed according to the standard operating procedures of the Clinic which was withdrawal of efavirenz and counselling.

## Results

### Demographics and clinical characteristics

**Fig. 1:**
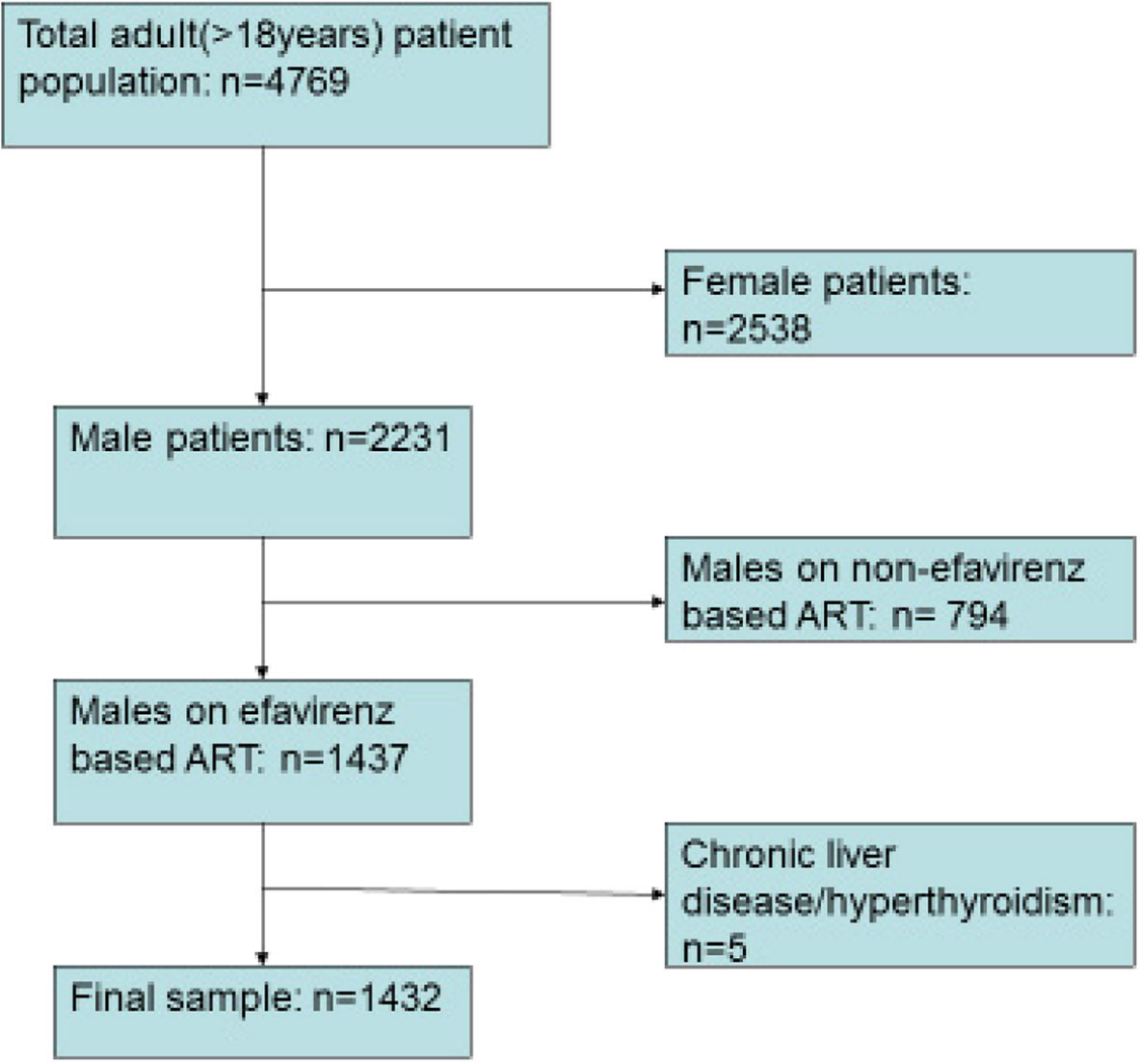
Flow chart showing selection of participants

We analysed data for 1432 HIV-positive men who started an efavirenz-based regimen between 2004 and March 2017 and followed up for 3298 years (median: 1.7 years; IQR: 1.0–2.6). Four patients with chronic liver disease and one with hyperthyroidism were removed according to the exclusion criteria to reach a number of 1432. Figure 1 shows the flow chart showing the selection of participants for analysis.

The median age at baseline was 40 years, (IQR: 33–48). Half of the participants (50%) were in WHO stage 1 at baseline. Median baseline CD4 count was 260 cells/mm^3^ (IQR: 126–412) whilst the median BMI was 21 kg/m^2^ (IQR: 19–25). Table 1 shows the baseline characteristics of the participants.

**Table 1:** Demographic and Clinical characteristics and outcomes of males on ART screened for gynecomastia (*N* = 1432)

Characteristic	All (*N* = 1432)	Gynecomastia (*n* = 73)
Demographics		
Age (years), median (IQR)	40 (38–48)	40 (33–48)
Baseline CD4 count median (IQR)	260 (126–412)	318 (192–469)
Baseline BMI, median (IQR)	21 (19–24)	21 (19–23)
Baseline WHO (%)		
Stage 1	548 (50)	28 (48)
Stage 2	217 (20)	14 (24)
Stage 3	233 (21)	10 (17)
Stage 4	97 (9)	6 (10)
Follow up time (years), median (IQR)	1.7 (1.0–2.6)	
Presentation of gynecomastia		
Unilateral		28 (38)
Bilateral		43 (59)
Unknown		2 (3)
Outcomes		
Complete resolution		28 (38)
Partial resolution		43 (59)
Non resolution		2 (3)
Unknown		5 (7)

*IQR* interquartile range, *WHO* World Health Organization Staging

### Gynecomastia

Among the 1432 adult men on efavirenz-containing ART, 73 (5%) developed gynecomastia. The incidence of gynecomastia was 22.1/1000 person-years (CI: 17.3–27.8). Among those that developed gynecomastia, the median baseline CD4 count was 318 cells/mm^3^ (IQR: 192–467) while the median baseline BMI was 21 kg/m^2^ (IQR: 19–23).

**Fig. 2:**
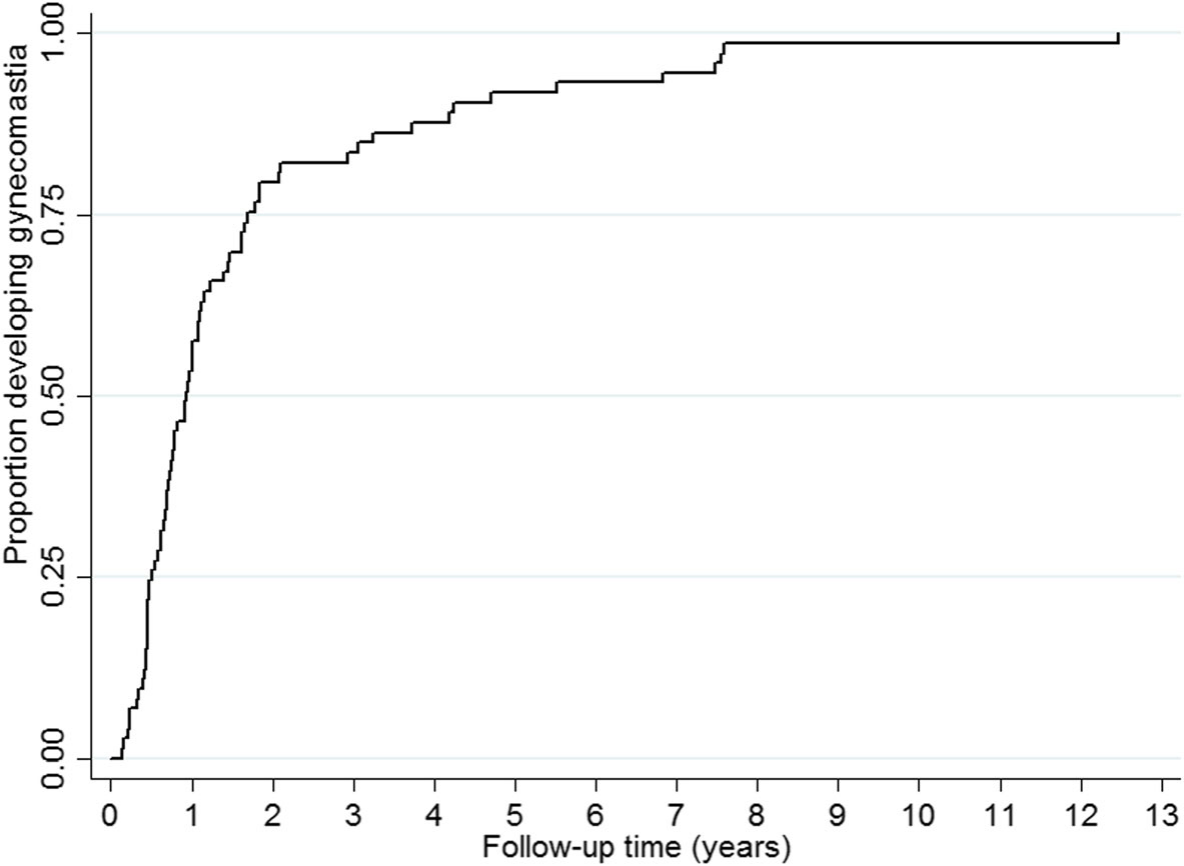
Kaplan-Meier curve (inverted) showing time to developing gynecomastia among cases

More than half of the gynecomastia cases were bilateral (59%), while 38% were unilateral, and 3% did not have documented complete description. Table 1(above) summarizes the characteristics of patients with gynecomastia. Fifty-three (80%) participants developed gynecomastia within the first two years of starting an efavirenz-containing regimen (Fig. 2).

### Associations with concomitant drugs

Our cohort comprised of males aged ≥18 years at efavirenz commencement. There was no association between age and concomitant exposure to isoniazid or amlodipine. None of the patients exposed to methyldopa or spironolactone developed gynecomastia. In the univariate analysis model including age (both as a continuous and categorical variable), isoniazid and amlodipine exposure, none of the three risk factors were significantly associated with developing gynecomastia (Table 2).

**Table 2:** Risk factor analysis

	Univariate Analysis		
Characteristic	HR	CI	*p*-value
Age			
18–24	–	–	–
> 24	0.7	0.38–1.54	0.52
Isoniazid	0.94	0.55–1.65	0.85
Amlodipine	0.43	0.11–1.75	0.25

### Outcomes

Among the 73 participants who developed gynecomastia, 61 (84%) resolved, four (5%) had partial resolution, whilst three (4%) did not resolve. Outcomes for five (7%) cases were unknown. Of those that resolved completely, two had no antiretroviral change while the rest stopped efavirenz. Amongst the 61 that resolved, 46 (75%) resolved within three months of stopping efavirenz. All the cases of partial resolution were in the age group 18–20 years.

## Discussion

Our results showed that gynecomastia was uncommon in this cohort, with most cases developing within two years of starting an efavirenz–based regimen. However, this was higher than in previous studies done elsewhere. The study demonstrated favorable outcomes upon stopping efavirenz.

The incidence of gynecomastia in our cohort was more than that of a study done in France which was 8/1000 patient years [12]. In Malawi, a study on 1207 men found a prevalence of 6% with evidence of adverse psychological effects [9]. The majority of the cases were bilateral and this is consistent with studies done in Sub-Saharan-Africa [3, 4, 10]. Most cases (83%) resolved upon stopping efavirenz and this is in keeping with findings from case studies done in Malawi, South Africa and Mozambique [3, 4, 7]. Median duration for clinical resolution in our study was 3 months after stopping the efavirenz. This is comparable to other studies that have reported resolution time of between 2 and 9 months [4, 6, 13, 15]. Rapid and complete resolution of gynecomastia is expected if the offending agent is withdrawn early. Newlands Clinic patients were counselled on the possibility of gynecomastia whilst taking efavirenz and clinicians actively looked for such side effects during routine visits. It was therefore likely that the adverse event was detected early, and withdrawal was timeous. If gynecomastia is present for more than a year, the glandular tissue is replaced by fibrosis and this can make reversal difficult [15]. Early recognition of gynecomastia may prompt early discontinuation of the offending agent with positive outcomes. With the ‘test and treat’ approach recommended by the 2017 WHO HIV guidelines [1], more patients will be put on an efavirenz = based ART regimen and differentiated care models for stable patients will see some patients being reviewed once or twice a year, and cases of gynecomastia may go unnoticed and unmanaged. Gynecomastia can potentially cause psychological harm and mitigate against the success of suppressive ART. In Malawi gynecomastia was associated with embarrassment and low self-esteem in more than half of the participants with a third of them not reporting it to their health care providers [9]. Male patients on ART should be closely monitored for gynecomastia during ART with prompt withdrawal of efavirenz in case of an adverse event.

All cases of partial resolution occurred in adolescents, and this may indicate that gynecomastia was due to other factors in addition to or besides efavirenz. Physiological hormonal changes could explain the development of gynecomastia though efavirenz could have contributed.

There were no associations found with other concomitant drugs such as spironolactone and amlodipine. Gynecomastia has been reported with high doses (400 mg/24 h) of spironolactone and in this cohort much lower doses were used (up to 100 mg/24 h) and this could explain the lack of association [8]. Cases of gynecomastia due to isoniazid were very rare [16, 17] and in our study we found no association even though this was a commonly prescribed drug.

The study is to the best of our knowledge, the first cohort study on incidence of gynecomastia among male patients taking an efavirenz -based regimen in Sub-Saharan Africa. However, our study has some limitations. It was a retrospective cohort analysis of routinely collected data, so the information was not collected according to strict study protocol. Diagnosis was based on a clinical finding of glandular proliferation beneath the areolar complex and no ultrasound was done, or hormone tests done to definitively diagnose gynecomastia. It was not possible to differentiate between efavirenz-related and physiological gynecomastia due to puberty in the younger age groups. In this study; only cases of patients at least 18 years of age were considered to minimize cases of physiological gynecomastia. However, people with HIV may have delayed puberty and hence misclassified [18]. Being a retrospective review; follow up information could not be obtained for all cases. Lastly, gynecomastia in this cohort was diagnosed after a patient self-reported enlarged breast, with no active screening done and therefore there is a possibility of underreporting.

Our results have important implications for treatment of HIV-infected males in low income countries where efavirenz still forms the backbone of first line ART. Gynecomastia has significant psychological adverse effects and may impact on treatment success. Clinicians should actively look for gynecomastia when patients come for review and manage it timeously. Patients should be counselled appropriately before starting efavirenz and encouraged to seek help on time.

## Conclusion

The incidence of gynecomastia in male patients on an efavirenz-based regimen was low with most cases developing in the first two years of starting treatment. Withdrawal of efavirenz had positive outcomes with most cases achieving complete resolution. Active screening and early withdrawal of efavirenz in patients who develop gynecomastia is recommended in order to improve the quality of life and promote adherence to antiretroviral therapy.

## Data Availability

The dataset used in this study are available from the corresponding authors.

## Abbreviations

AIDS: Acquired immunodeficiency syndrome
ART: Antiretroviral therapy
BMI: Body mass index
CD4: Cluster Differentiation
CI: Confidence interval
HIV: Human immunodeficiency virus
HR: Hazard ratio
IQR: Interquartile range
MRCZ: Medical Research Council of Zimbabwe
STATA: Statistics and data
WHO: World Health Organisation
